# Development of Chrome-Doped Hydroxyapatite in a PVA Matrix Enriched with Amoxicillin for Biomedical Applications

**DOI:** 10.3390/antibiotics14050455

**Published:** 2025-04-30

**Authors:** Steluta Carmen Ciobanu, Daniela Predoi, Simona Liliana Iconaru, Krzysztof Rokosz, Steinar Raaen, Coralia Bleotu, Mihai Valentin Predoi

**Affiliations:** 1 National Institute of Materials Physics, Atomistilor Street, No. 405A, P.O. Box MG 07, 077125 Magurele, Romaniasimonaiconaru@gmail.com (S.L.I.); 2 Faculty of Electronics and Computer Science, Koszalin University of Technology, Śniadeckich 2, PL 75-453 Koszalin, Poland; rokosz@tu.koszalin.pl; 3 Department of Physics, Norwegian University of Science and Technology (NTNU), Realfagbygget E3-124 Høgskoleringen 5, NO 7491 Trondheim, Norway; 4 Department of Cellular and Molecular Pathology, Stefan S. Nicolau Institute of Virology, 030304 Bucharest, Romania; cbleotu@yahoo.com; 5 Research Institute of the University of Bucharest (ICUB), University of Bucharest, 060023 Bucharest, Romania; 6 The Academy of Romanian Scientist, 050711 Bucharest, Romania; 7 Department of Mechanics, University Politehnica of Bucharest, BN 002, 313 Splaiul Independentei, Sector 6, 060042 Bucharest, Romania

**Keywords:** chrome, hydroxyapatite, morphology, coatings, chemical composition, biocompatibility, antimicrobial activity, PVA

## Abstract

**Background/Objectives**: In this paper, we report the development of the first chrome-doped hydroxyapatite in a poly (vinyl alcohol) (PVA) matrix enriched with amoxicillin for biomedical applications. The development of chromium-doped hydroxyapatite coatings in a PVA matrix enriched with amoxicillin aims to provide new biomaterials with improved physico-chemical and biological properties, making them promising candidates for biomedical applications. **Methods:** Through ultrasound studies, we obtained valuable information on the stability of the samples. X-ray diffraction (XRD), X-ray photoelectron spectroscopy (XPS), scanning electron microscopy (SEM), Fourier-transform infrared (FTIR) spectroscopy, metallographic microscopy (MM), and atomic force microscopy (AFM) were employed for the characterization of the samples. The biocompatibility of the CrHApAPV and CrHApAPV-Ax coatings was assessed using the MG63 human osteoblast-like cell line. To evaluate the cytotoxic potential of these coatings, the cell viability was quantified using the MTT assay after 24 h of incubation. The antibacterial activity of the coatings was evaluated with the aid of the reference strain *Pseudomonas aeruginosa* ATCC 27853 (*P. aeruginosa*). **Results**: The XRD patterns of CrHApAPV and CrHApAPV-Ax samples were examined to evaluate the effects of PVA and amoxicillin on the lattice parameters, unit cell volume, and average crystallite sizes. The results of the in vitro antibacterial assay demonstrated that both the CrHApAPV and CrHApAPV-Ax coatings exhibited very good antibacterial properties for all the tested time intervals. **Conclusions:** Our results underline the stability of the analyzed samples. Moreover, our physico-chemical and biological studies highlight that CrHApAPV and CrHApAPV-Ax coatings could be considered promising materials for biomedical uses.

## 1. Introduction

Hydroxyapatite (HAp), with the chemical formula Ca_10_(PO_4_)_6_(OH)_2_, is one of the most extensively used materials in the field of biomaterials. As a representative calcium phosphate compound, it exhibits a chemical composition that closely resembling the mineral phase of bone and other hard human tissues. Due to its structural properties, it is possible to make controlled adjustments to the physicochemical and biological features of hydroxyapatite [1,2]. The crystalline structure of HAp contains two distinct calcium sites (Ca^2+^) that allow ionic substitutions that significantly influence the crystal’s physico-chemical properties [3]. Various studies have focused on HAp doping with foreign divalent cations such as Mg^2^⁺ and Zn^2^⁺ [4,5,6]. Additionally, the incorporation of trivalent ions like Cr^3^⁺ and Eu^3^⁺ into the HAp has been explored as a strategy for tuning its bulk properties [7,8,9,10,11]. It is well known that chromium is an essential trace element that can be found in human body; however, it can exhibit toxic effects at high concentrations [10,11]. Chromium exists in four primary oxidation states, Cr(0), Cr(II), Cr(III), and Cr(VI), with Cr(III) and Cr(VI) being the most stable and naturally abundant [10,11]. Cr(III) is generally less cytotoxic than Cr(VI) due to its lower solubility and limited cellular uptake [12]. On the other hand, in the recent years, inorganic/organic composite systems have attracted significant attention as potential candidates for drug delivery carriers for bone tissue treatment. HAp has been extensively used in bone tissue engineering applications due to its excellent bioactivity and osteoconductive properties [13,14,15,16]. Various studies have focused on the development of inorganic–organic composites, particularly through the crystallization of the inorganic phase, mediated by organic compounds. Various attempts using polymers such as collagen [17], poly (acrylic acid) [18], poly (lactic acid) [19], poly (vinyl alcohol) (PVA) [20], and chitosan [21] have been reported in the literature. These polymers offer a wide range of possibilities depending on their chemical composition and molecular structure. Poly (vinyl alcohol) (PVA) is a hydrophilic polymer known for its excellent physico-chemical properties, as well as its biocompatibility, which has contributed to its use as a biomaterial in various applications [22,23]. Various methods have been reported in the literature regarding the preparation of HAp/PVA composites, including the fabrication of composite scaffolds [24], coatings [25], composite gels [26], and polymer-coated ceramics [27]. These kinds of materials were employed in a range of biomedical applications such as bone substitutes, in hard tissue repair, and as drug delivery systems [13].

L. Stipniece et al. [13] reported the successful preparation of uniform spherical microgranules composed of nanosized hydroxyapatite crystallites embedded in poly (vinyl alcohol) (PVA) matrices through in situ precipitation and spray drying methods. Their study demonstrated the development of functionalized hydroxyapatite/PVA composites for potential applications in bone tissue engineering, drug delivery, and injectable bone fillers, providing a novel approach for producing moldable materials with the desired physico-chemical and biological properties for biomedical use [13].

Another study conducted by A.S. Asran and coworkers [28] developed a new PVA/Col/n-HAp biocomposite nanofiber scaffold that mimics the nanostructure and composition of bone, with aligned n-HAp crystals along the PVA/Col fibers. The obtained scaffold has controllable porosity and improved mechanical properties, and the best results were obtained with the 5 wt% n-HAp, making it suitable for non-load-bearing bone tissue engineering and bioactive bone graft applications [28]. In the work conducted by S.A. Poursamar and collaborators [29], they produced organic (PVA) and inorganic (HAp) phases in nanocomposite scaffolds. In vitro tests with osteoblast cells confirmed the scaffolds’ biocompatibility, with cells successfully attaching to the scaffold pores, highlighting their potential uses in bone repair applications due to the improved cell response, biocompatibility, and mechanical properties [29].

Furthermore, a recent study by C.S. Ciobanu et al. [30] demonstrated that CrHAp (x_Cr_ = 0.05) and CrHApAx coatings, obtained using the dip coating technique, exhibited good stoichiometry, stability, and biological properties. The presence of hydroxyapatite and amoxicillin was confirmed through various analyses, including XPS, XRD, FTIR, and SEM [30]. The CrHApAx coating showed superior antibacterial activity against *Pseudomonas aeruginosa* compared to CrHAp, and both coatings displayed excellent biocompatibility with MG63 cells, indicating their potential for uses in bone tissue engineering and implant applications [30]. Their results suggest that these biomaterials effectively combine biocompatibility with antibacterial properties, making them promising candidates for biomedical use [30].

In this context, the development of new coatings based on chrome-doped hydroxyapatite in a PVA matrix enriched with amoxicillin could be a novel approach for developing new materials for biomedical applications by producing coatings with biocompatibility, osteoconductivity, and enhanced antibacterial properties. These coatings could be suitable candidates for various applications in the medical domain such as in bone implants, tissue engineering, drug delivery, etc.

In this work, we report the development of coatings consisting of chrome-doped hydroxyapatite in a polyvinyl alcohol matrix (CrHApAPV) and chrome-doped hydroxyapatite in a polyvinyl alcohol matrix enriched with amoxicillin (CrHApAPV-Ax) using the spin coating technique. Non-destructive ultrasound (US) measurements were used to obtain data about the samples’ stability. Moreover, the new developed coatings were subjected to physico-chemical and biological characterization.

## 2. Results

This study explored the physicochemical and biological characteristics of CrHApAPV and CrHApAPV-Ax coatings created using the sol-gel technique. These coatings were derived from dense aqueous suspensions of CrHApAPV and CrHApAPV-Ax. A key focus of the research was the stability of these dense suspensions, as it plays a pivotal role in producing coatings suitable for bone implants. Ultrasound measurements were utilized to assess the stability of these concentrated suspensions. Suspension stability is vital for coating effectiveness because it ensures the uniform distribution of particles within the suspension. This uniformity directly impacts the coating’s properties, such as adhesion, durability, and appearance. If the suspension is unstable, the particles may aggregate or settle, leading to an uneven coating, reduced performance, and compromised functionality [31]. Stability also plays a role in maintaining the physicochemical properties of the coating during storage and application [32].

**Figure 1:**
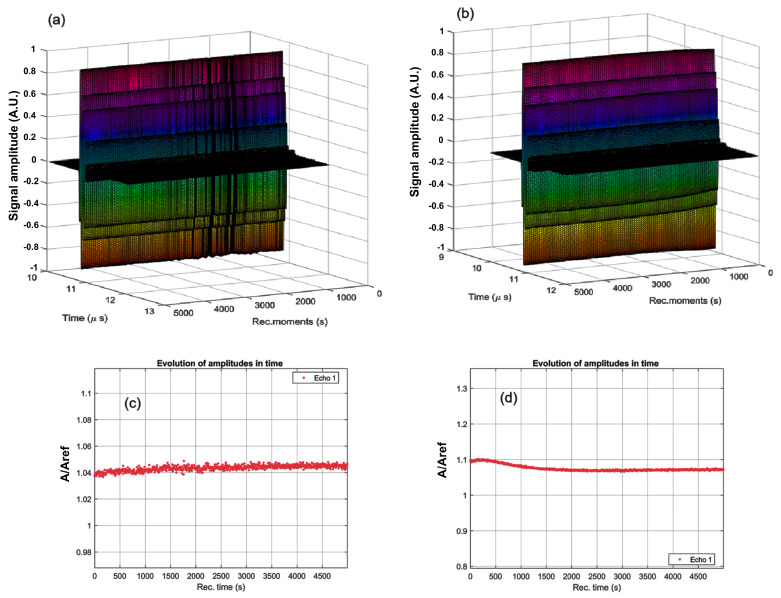
Evolution of the recorded signals over 5000 s for the CrHApAPV (**a**) and CrHApAPV-Ax (**b**) samples. The recorded signal amplitudes during the experiment for CrHApAPV (**c**) and CrHApAPV-Ax (**d**).

A superposition of the 1000 registered signals, recorded every 5 s using a digital oscilloscope, for the CrHApAPV and CrHApAPV-Ax samples is shown in Figure 1a,b. From right to left are all the signals covering the 5000 s of the process evolution. It can be observed that in the case of the CrHApAPV sample, the signals were not uniform, with temporary variations in the acoustic signal amplitudes, which are visible in the plot. For the CrHApAPV-Ax sample, the sedimentation process was very slow, with imperceptible variations in the acoustic signal amplitudes (Figure 1b). The evolution of the amplitudes over time is detailed in Figure 1c,d. A slow and continuous increase in amplitude was observed during the 5000 s monitoring period for the CrHApAPV sample (Figure 1c). It can be observed that the ultrasonic signal generally had an amplitude A/Aref > 1, which was attributed to the presence of nanoparticles with lower attenuation and a higher compressibility coefficient (Figure 1c). On the other hand, for the CrHApAPV-Ax sample, the sedimentation process was very slow, with imperceptible variations in the acoustic signal amplitudes in Figure 1d. It was observed that for the CrHApAPV-Ax sample, there was a slow increase in amplitude during the first 200 s, followed by a reduction in amplitude during the next 2000 s, and finally, a slow increase in the signal’s peak amplitude. It can be observed that the ultrasonic signal generally had an amplitude larger than in that of the reference fluid (A/Aref > 1), which was attributed to the presence of nanoparticles with low attenuation and a higher compressibility (Figure 1d).

**Figure 2:**
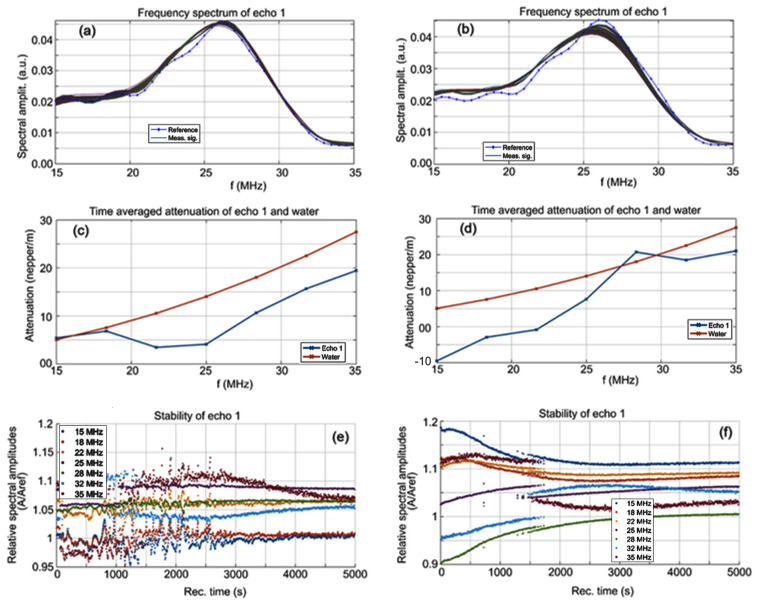
Spectral amplitudes of all recorded signals for CrHApAPV (**a**) and CrHApAPV-Ax (**b**) samples. Time averaged attenuation for the investigated frequency range for CrHApAPV (**c**) and CrHApAPV-Ax (**d**) samples. Relative spectral amplitudes vs. time for CrHApAPV (**e**) and CrHApAPV-Ax (**f**) samples.

The evolution of the frequency spectrums for each of the 1000 signals for the CrHApAPV and CrHApAPV-Ax samples is shown in Figure 2a,b and for comparison, the spectrum of the reference liquid (double distilled water, in dotted blue line) is also plotted. It can be seen that the spectrums were close to the spectrum of the reference liquid, indicating that the elastic properties of the nanoparticles are similar to those of the reference liquid and that the suspension has a very high stability (Figure 2a,b). The average amplitudes in the frequency spectrum over the 1000 spectrums are shown in Figure 2c,d along with the natural logarithm of the amplitude ratio of the samples to the reference liquid. The CrHApAPV (Figure 2c) suspension was very similar to the reference liquid at the lowest frequencies (15–18 MHz), whereas at the higher frequencies, the attenuation was lower than that of the reference liquid, confirming the presence of nanoparticles with low intrinsic attenuation and a higher compressibility coefficient. The CrHApAPV-Ax (Figure 2d) suspension was also similar to the reference liquid. At the lowest (15–25 MHz) and highest (32–35 MHz) frequency ranges, the attenuation was lower than that of the reference liquid, which indicates the presence of solid nanoparticles immersed in the suspension. At a frequency of 28 MHz, there was a close similarity in the amplitudes of the CrHApAPV-Ax sample and reference liquid. More insights can be obtained by analyzing the spectral stability, representing the amplitude of the frequency component of each spectrum, as function of time (Figure 2e,f).

Some remarkable features were observed for the CrHApAPV sample (Figure 2e). During the first 1100 s, most of the spectral components of the signals exhibited amplitude variations but only after roughly 3000 s from the start of the experiment did all the spectral amplitudes resume a steady evolution over time. During the intermediary period between 1100 and 3000 s, the passage of various clusters in front of the transducers and the rapid evolution of the suspension components led to erratic behavior, which is typical for this CrHApAPV suspension. For the CrHApAPV-Ax sample (Figure 2f), it was noted that after the first 1200 s, the sedimentation superior layer slowly passed in front of the transducers. The concentration of the various nanoparticles in the suspension changed considerably at about 500 s. Consequently, the spectral amplitudes of the CrHApAPV-Ax suspension exhibited small decreases (e.g., at 18 MHz) or increases (e.g., 22 or 28 MHz) during this time interval. These features reflect the variability in the behavior of the various types of nanoparticles in the CrHApAPV-Ax suspension.

We determine also a quantitative stability parameter as being slope of the amplitude dependency on time, shown on Figure 1c,d. The CrHApAPV sample is considered to be very stable ($S=\bar{\frac{dA}{Adt}}$= 3.26·10^−6^ s^−1^) while the CrHApAPV-Ax sample is considered to be stable ($S=\bar{\frac{dA}{Adt}}$= 2.69·10^−5^ s^−1^) in which *A* is the signal amplitude, with a bar above indicating time averaging, very close to *S* = 0 for the reference liquid.

**Figure 3:**
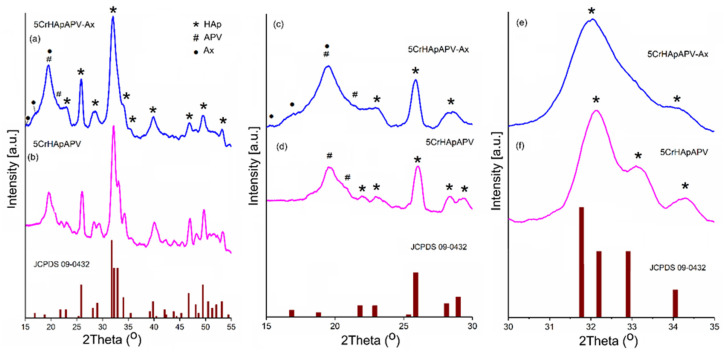
XRD patterns of CrHApAPV-Ax (**a**) and CrHApAPV (**b**) coatings in the 2θ range of 15° to 55°. XRD patterns of CrHApAPV-Ax (**c**) and CrHApAPV (**d**) coatings in the 2θ range of 15° to 30°. XRD patterns of CrHApAPV-Ax (**e**) and CrHApAPV (**f**) coatings in the 2θ range of 30° to 35°. XRD patterns of pure hexagonal hydroxyapatite JCPDS 09-0432 (burgundy columns).

The evaluation of the CrHApAPV-Ax and CrHApAPV coatings was conducted using X-ray diffraction (XRD) analysis to determine their structural properties and average crystallite sizes. The diffraction patterns for these coatings are showcased in Figure 3a,b for the 2θ range of 15° to 55°. More detailed patterns in narrower ranges of 15° to 30° and 30° to 35° are depicted in Figure 3c–f. For comparison, the XRD patterns for the pure hexagonal hydroxyapatite (standard JCPDS card 09-0432) are also included in Figure 3. The XRD analysis of the CrHApAPV and CrHApAPV-Ax samples (Figure 3a,b) revealed characteristic peaks that align with the hexagonal structure of pure hydroxyapatite (HAp), consistent with JCPDS # 09–0432. Notable diffraction planes observed included (200), (111), (002), (102), (210), (211), (300), (202), (310), (222), (213), and (004). These results confirmed the predominant HAp phase with a P63/m space group.

For the CrHApAPV sample (Figure 3b,d), APV-specific peaks at 2θ angles of 19.63° and 20.89° were observed. In the CrHApAPV-Ax sample (Figure 3a,c), apart from the predominant HAp phase and APV-specific peaks, additional peaks related to amoxicillin were detected at 2θ angles of 15.02°, 17.08°, and 19.5°. Notably, these peaks were broader, which indicates possible changes in the crystallite size or strain in the crystal lattice. A decrease in the intensity of the peaks was observed for the CrHApAPV-Ax sample, suggesting alterations in the phase composition or reduced crystallinity.

The XRD patterns of the CrHApAPV and CrHApAPV-Ax samples were analyzed to investigate the impact of the presence of PVA and amoxicillin on the lattice parameters, unit cell volume, and average crystallite sizes. In this study, four diffraction peaks corresponding to the hydroxyapatite (HAp) crystal structure were analyzed. These peaks are associated with the Miller indices (002), (211), (112), and (300). The 2θ values for these peaks were accurately determined using the least squares method implemented through a fitting program with a pseudo-Voigt function. This precise analytical approach ensures the reliable estimation of the diffraction peak positions. The values presented in Table 1 demonstrated a decrease in the average crystallite size when amoxicillin was present in the sample (CrHApAPV-Ax).

The c/a ratio of CrHApAPV, which was approximately 0.731, reflects the structural arrangement of its hexagonal crystal lattice. The c/a ratio of 0.734 in CrHApAPV-Ax reflects subtle variations in the lattice dimensions of its hexagonal crystal structure. This value indicates that the unit cell is slightly elongated along the c-axis compared to the a-axis, which is characteristic of hydroxyapatite’s hexagonal symmetry (space group P63/m). This c/a ratio of 0.734 could result from various factors such as temperature, pH, and precursor concentrations during the synthesis of Hap, which could subtly impact the crystallite size and the lattice dimensions. On the other hand, the incorporation of amoxicillin (C_16_H_19_N_3_O_5_S) led to an increase in the c/a ratio. Gafurov et al. [33] showed that N can be substituted in HAp as NO_3_^−^. Building on this, Biktagirove et al. [34] demonstrated the versatility of NO_3_^−^ placement within the HAp structure, noting that it could occupy both the A-site, replacing hydroxyl groups (OH^−^), and the B-site, substituting for phosphate groups (PO_4_^3−^). These findings highlight the structural adaptability and potential for functional modification in HAp.

**Figure 4:**
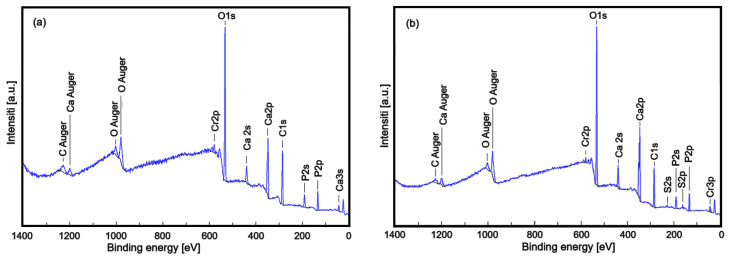
Full XPS spectra of CrHApAPV (**a**) and CrHApAPV-Ax (**b**) samples.

Through XPS analysis, the study identified the elemental composition and chemical changes in the chromium-doped and amoxicillin-enriched hydroxyapatite in a PVA matrix. The XPS spectra of the CrHApAPV and CrHApAPV-Ax samples (Figure 4) showed peaks for carbon (C), oxygen (O), calcium (Ca), phosphorus (P), and chromium (Cr). However, nitrogen (N) and sulfur (S) only appeared in the CrHApAx sample, confirming the incorporation of amoxicillin.

**Figure 5:**
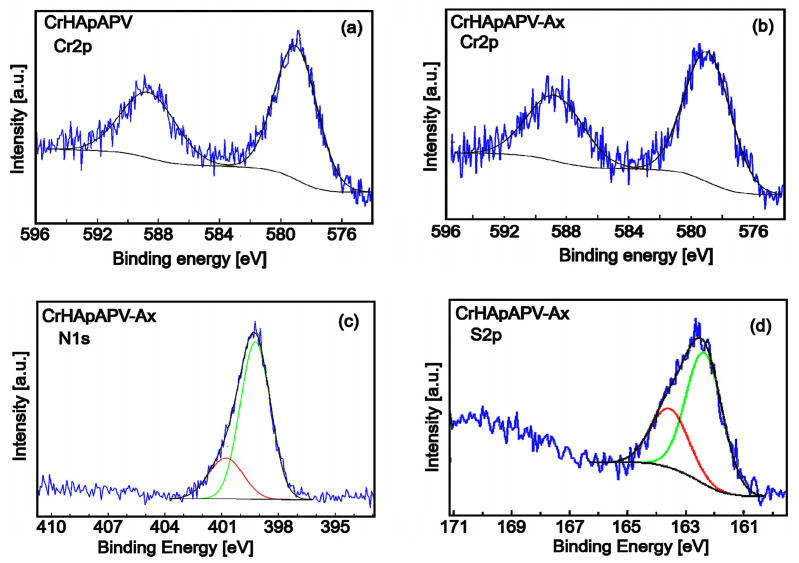
High-resolution XPS spectra of Cr 2p for CrHApAPV (**a**) and CrHApAPV-Ax (**b**) samples. High-resolution XPS spectra of N1s (**c**) and S 2p (**d**) for CrHApAPV-Ax samples.

Figure 5 presents the high-resolution XPS spectra of Cr2p, N1s, and S2p. Figure 5 a,b show the high-resolution Cr2p spectra for CrHApAPV and CrHApAPV-Ax. The Cr2p spectra for both samples were fitted with the characteristic doublets 2p3/2 and 2p1/2, which were separated by approximately 9.6 eV and had an area ratio close to 2:1 (as shown in Figure 5a,b). Consistent with prior studies [35], the high-resolution spectra for Cr2p revealed Cr-2p 3/2 peaks centered within 574–582 eV and Cr-2p 1/2 peaks in the range of 583–592 eV, indicating the presence of three distinct chromium valence states. Previous research has demonstrated that the pairs of Cr-2p 3/2 and Cr-2p 1/2 peaks located at (577.6 and 587.2 eV), (576.5 and 585.5 eV), and (579.1 and 589.7 eV) are associated with Cr (III) in Cr_2_O_3_ [36,37], Cr (IV) in CrO_2_ [38,39], and Cr (VI) [40] in CrO_3_, respectively.

The high-resolution XPS spectra of N1s for the CrHApAPV-Ax sample (Figure 5c) after deconvolution showed two components. The first component was located at a binding energy (BE) of 399.21 eV, which is specific to C-N and N-H bonds. The second component was observed at a BE of 400.71 eV, which showed a more aggressive electron loss and possibly the appearance of bonds with O. The observed peaks are in agreement with those of previous studies [41]. The high-resolution XPS spectrum of S2p for the CrHApAPV-Ax sample was fitted with the specific doublet 2p3/2 (162.35 eV) and 2p1/2 (163.55 eV), spaced at approximately 1.2 eV and with an area ratio close to 2:1 (Figure 5d). The binding energy indicated the formation of sulfate. This result is in agreement with that of previous studies [42,43].

**Figure 6:**
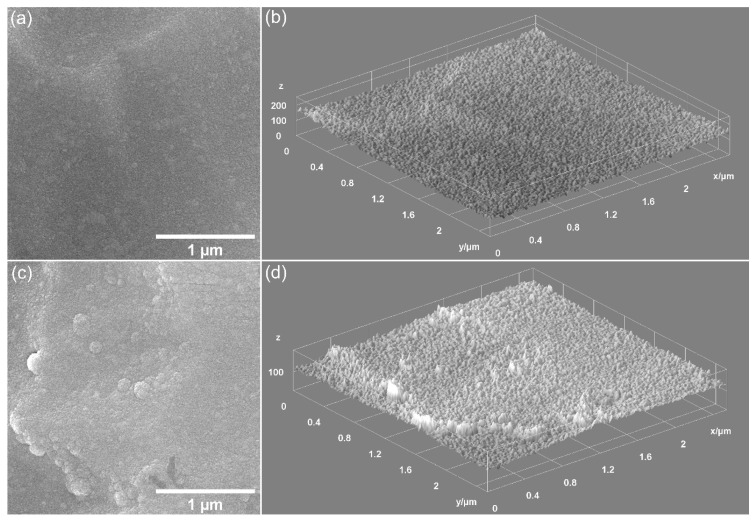
Two- and three-dimensional SEM images of CrHApAPV (**a**,**b**) and CrHApAPV-Ax (**c**,**d**) coatings.

Preliminary data on the surface morphology of the CrHApAPV and CrHApAPV-Ax coatings produced using the spin coating method were obtained (SEM measurements). Figure 6 shows the 2D and 3D SEM images obtained for the CrHApAPV and CrHApAPV-Ax coatings deposited on Si.

The 2D SEM images obtained for the CrHApAPV coating suggested the presence of a homogeneous and continuous deposited surface. Another notable characteristic of the surface morphology of the CrHApAPV coating, as revealed by the SEM analysis, was the presence of agglomerated nanoparticles distributed across the examined surface. Also, the SEM images underlined the absence of notable surface defects such as cracks. The 2D SEM images obtained for the CrHApAPV-Ax coating, showed a significant improvement in surface uniformity and homogeneity. The SEM results indicated a more consistent distribution of agglomerated nanoparticles across the analyzed area. The 3D SEM images shown in Figure 6b,d clearly confirmed the results of the 2D SEM images. Therefore, the 3D SEM images obtained for the surfaces of the CrHApAPV and CrHApAPV-Ax coatings highlighted the presence of a continuous, homogeneous, and uniform coating. The slight morphological changes observed in the case of the CrHApAPV-Ax coating can be attributed to the presence of amoxicillin in the sample. Furthermore, these results are in good agreement with previously reported results [30].

Preliminary data on the thickness of the CrHApAPV and CrHApAPV-Ax coatings were obtained by performing SEM transverse cross-section studies (Table 2).

Thus, our results suggest that the thickness of the CrHApAPV coating was 175 ± 10 nm, while the thickness of the CrHApAPV-Ax coating was 188 ± 10 nm.

**Figure 7:**
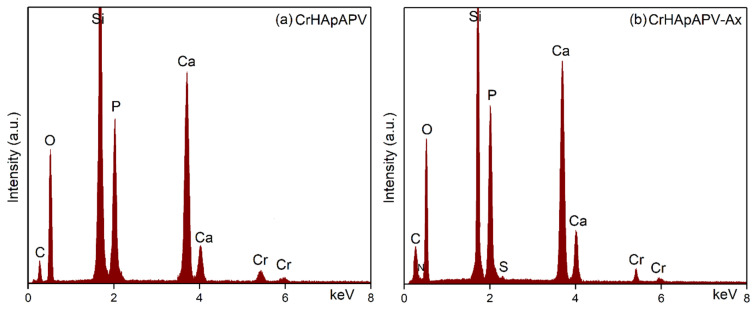
EDS spectra of CrHApAPV (**a**) and CrHApAPV-Ax (**b**) coatings.

Data on the chemical composition of the CrHApAPV and CrHApAPV-Ax coatings were obtained by performing EDS studies. Figure 7 shows the EDS spectra for the CrHApAPV and CrHApAPV-Ax coatings.

The EDS spectra presented in Figure 7 correspond to the CrHApAPV and CrHApAPV-Ax coatings and provide essential insights into their elemental composition and purity. In the case of the CrHApAPV sample (Figure 7a), the EDS spectrum displays distinct lines specific to calcium (Ca), phosphorus (P), oxygen (O), and chromium (Cr), consistent with the expected elemental constituents of CrHApAPV. Moreover, the EDS spectrum of the CrHApAPV-Ax coating (Figure 7b) also confirmed the presence of the base matrix elements (CrHApAPV: Ca, P, O, and Cr) and revealed additional lines that are specific to nitrogen (N) and sulfur (S). These two new elements (N and S) are from the amoxicillin, thereby confirming its successful integration within the coating. The intense silicon (Si) line observed in both EDS spectra was from the silicon substrate used for coating deposition. Furthermore, in the obtained EDS spectra, there are no additional/supplementary lines, attesting to the purity of the CrHApAPV and CrHApAPV-Ax coatings.

**Figure 8:**
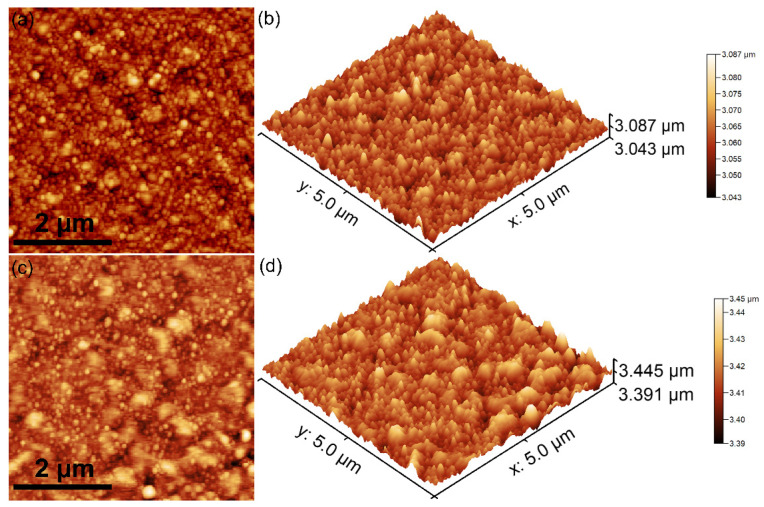
Two-dimensional AFM topographies of CrHApAPV (**a**) and CrHApAPV-Ax (**c**) coatings’ surfaces for an area of 5 × 5 µm^2^ and their corresponding 3D representations (**b**,**d**).

The morphological characteristics of the CrHApAPV and CrHApAPV-Ax coatings’ surfaces were assessed using AFM. The AFM surface topographies of the CrHApAPV and CrHApAPV-Ax coatings recorded on area with a size of 5 × 5 μm^2^ are depicted in Figure 8.

The AFM data revealed that the CrHApAPV coating exhibited a surface with a continuously and uniformly deposited layer with a smooth and homogeneous morphology. Both the 2D and 3D AFM images show the presence of evenly distributed nanometric particle agglomerates across the surface. Also, the AFM images show that there were no significant irregularities observed on the surface of the coatings. These results indicate a stable and consistent deposition of the CrHApAPV coating. On the other hand, the AFM analysis of the CrHApAPV-Ax coating showed that there was a notable enhancement in the surface uniformity and homogeneity. The 2D AFM images show a uniformly deposited layer with clearly visible particle agglomerates that appeared to be more pronounced and evenly distributed compared to the CrHApAPV coating. The 3D surface representation further confirmed this observation, emphasizing the continuous nature of the coating. These changes could be attributed to the presence of amoxicillin and suggests that the incorporation of amoxicillin influences the surface morphology by promoting the formation of a homogeneous and compact layer structure while encouraging the formation of uniformly distributed particle clusters. Details about the roughness of the surfaces were also determined by quantifying the roughness parameter R_RMS_. The roughness parameters (R_RMS_) for the CrHApAPV and CrHApAPV-Ax coatings were also calculated for the entire surface area. The values obtained for the roughness parameter R_RMS_ were 12.61 nm in the case of CrHApAPV and 17.45 nm for CrHApAPV-Ax.

**Figure 9:**
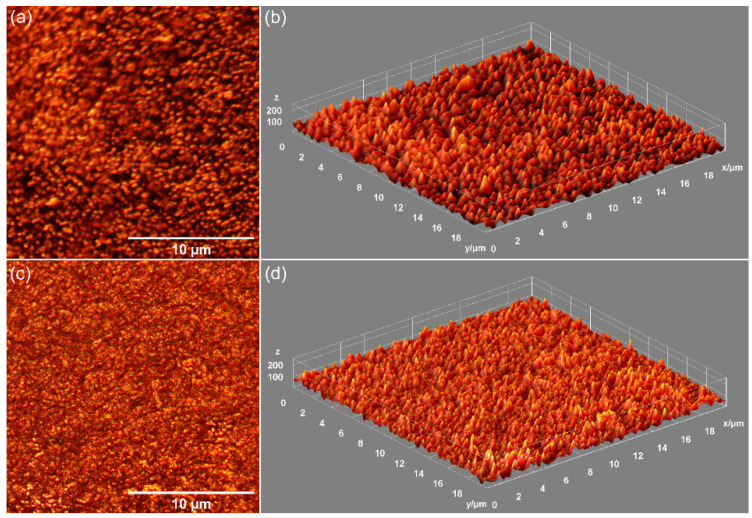
Two- and three-dimensional MM images of CrHApAPV (**a**,**b**) and CrHApAPV-Ax (**c**,**d**) coatings.

Additional valuable insights into the morphological characteristics of the CrHApAPV and CrHApAPV-Ax coatings’ surfaces were obtained through metallographic microscopy studies. The 2D metallographic images of the CrHApAPV and CrHApAPV-Ax coatings, captured at 20× magnification using a metallographic microscope (MM), are shown in Figure 9a,c. Furthermore, a 3D representation of the same surfaces is shown in Figure 9b,d.

The metallographic microscopy (MM) observations showed that both the CrHApAPV and CrHApAPV-Ax coatings’ surfaces were free from fissures or cracks. Furthermore, the 3D representation derived from the 2D metallographic images confirmed that the surface of the CrHApAPV and CrHApAPV-Ax coatings exhibited the characteristics of a smooth, continuous, and uniform coating. Also, in the 2D and 3D MM images, the presence of agglomerated nanoparticles on the coating’s surfaces was observed. In the case of the CrHApAPV-Ax coating, a more pronounced distribution of agglomerated nanoparticles was observed in the studied area, probably due to the presence of amoxicillin in the sample. These findings are consistent with the SEM analysis results, which further corroborates that the surface of the CrHApAPV and CrHApAPV-Ax coatings’ surfaces were free of significant defects, such as cracks or fissures. Therefore, the MM, AFM, and SEM results provide strong evidence of the coating’s homogeneity, continuity, and uniformity.

**Figure 10:**
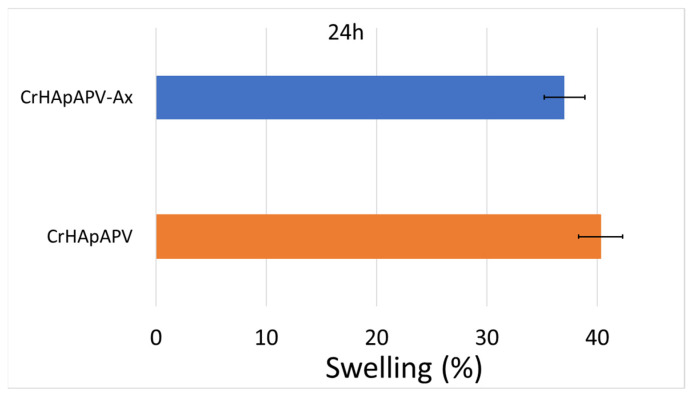
Swelling percentage of CrHApAPV and CrHApAPV-Ax coatings.

Figure 10 shows the results of the swelling test. After 24 h of soaking in the PBS solution, the swelling percentage for the CrHApAPV coating was around 40%. On the other hand, the swelling percentage obtained for the CrHApAPV-Ax coating after 24 h of soaking in the aqueous solution was about 37%.

According to the studies conducted by J. Prakash and collaborators [44], this behavior could be attributed to the presence of hydroxyapatite and amoxicillin in the analyzed samples, as they could block the pores of PVA. Similar results were previously reported in the studies conducted by N. Asy-Syifa et al. [45] and P. Chocholata et al. [46,47]. These results suggest that such coatings could help prevent the growth and development of bacteria, thus reducing the risk of infections [44]. Therefore, our results are in good agreement with those of J. Prakash, et al. [44].

**Figure 11:**
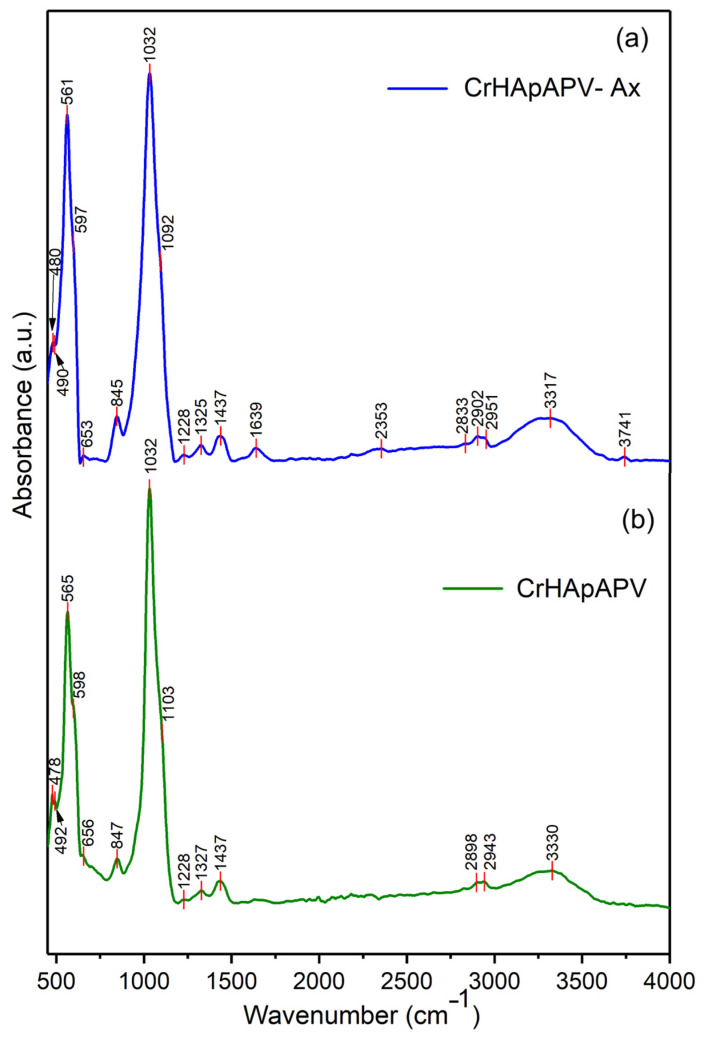
FTIR spectra of CrHApAPV-Ax (**a**) and CrHApAPV (**b**) in range between 450 and 4000 cm^−1^.

Figure 11 presents the general FTIR spectra in the 450–4000 cm^−1^ spectral domain for the CrHApAPV and CrHApAPV-Ax coatings. The FTIR spectra of CrHApAPV collectively confirmed the presence of specific functional groups from HAp (mainly maxima associated with the vibration of phosphate and hydroxyl groups), as well as the characteristic features of the PVA matrix (represented by maxima specific for O–H stretching vibrations, C–H stretching vibrations, the bending vibrations of CH_2_ groups, C–O stretching, and C–C stretching vibrations). Furthermore, in the case of CrHApAPV-Ax, among the functional group bands, there were bands from amoxicillin (presence of maxima associated with the vibration of C=O (β-lactamic ring) and vibration of carbonyl (-C=O) groups).

The general FTIR spectra of both analyzed samples confirmed the presence of functional groups characteristic of HAp. For the phosphate group (PO_4_^3^⁻), the following infrared absorption modes were observed: the symmetric P–O bending mode (ν_2_, 470–480 cm⁻^1^), the triply degenerate antisymmetric P–O stretching mode (ν_3_, 1040–1090 cm⁻^1^), and the triply degenerate antisymmetric P–O bending mode (ν_4_, 600–560 cm⁻^1^) [30,48,49]. The maxima that appear in the FTIR spectra due to the presence of PVA can be observed between 2900–2950 cm⁻^1^ (C–H stretching vibrations of aliphatic methylene (–CH_2_–) groups), 1740–1750 cm⁻^1^ (due to C=O stretching), 1420–1470 cm⁻^1^ (due to bending vibrations of CH_2_ groups), 1090–1140 cm⁻^1^ (due to C–O stretching and C–O–C asymmetric stretching; these vibrations are typical for the PVA polymer chain), and 840–880 cm⁻^1^ (due to C–C stretching vibrations) [49,50]. Usually, the broad maxima observed between 3300 and 3800 cm⁻^1^ are attributed to O–H stretching vibrations [49,50]. In the case of the CrHApAPV-Ax sample, the presence of the amoxicillin was highlighted by the presence of a weak maximum between 1630 and 1800 cm^−1^, which is specific for carbonyl (-C=O) vibration and the C=O vibration of the β-lactamic ring in amoxicillin [30,48]. All these vibration modes can be observed in Figure 11.

**Figure 12:**
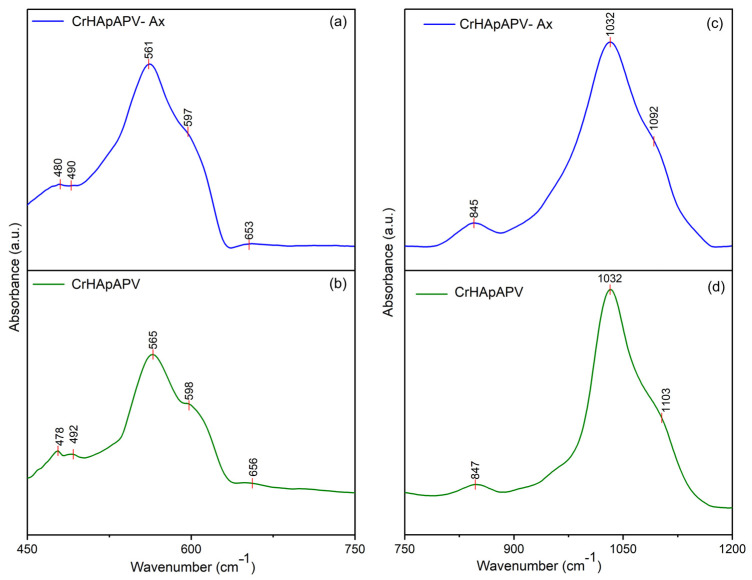
FTIR spectra of CrHApAPV-Ax in 450–700 cm^−1^ and 750–1200 cm^−1^ spectral domains (**a**,**c**). FTIR spectra of CrHApAPV in 450–700 cm^−1^ and 750–1200 cm^−1^ spectral domains (**b**,**d**).

Figure 12 presents the FTIR spectra of CrHApAPV-Ax and CrHApAPV recorded in two distinct spectral regions: 450–700 cm⁻^1^ and 750–1200 cm⁻^1^. For CrHApAPV, the weak maximum observed at 478 cm^−1^ and 482 cm^−1^ can be attributed to the ν_2_ vibration of phosphate group from HAp. The two clear bands that appear at 565 cm^−1^ and 597 cm^−1^ arose due to the ν_4_ vibration of phosphate group from HAp [30,48]. The maximum observed in the 750–1200 cm⁻^1^ region can be attributed to the vibrational modes of functional groups present in both PVA and HAp [30,48,49,50]. The weak maximum centered at 847 cm^−1^ correspond to C–C stretching vibrations from PVA [49,50]. Moreover, the intense maximum observed at 1032 cm^−1^ is due to the ν_3_ vibration of phosphate groups [30,48]. On the other hand, the maximum observed between 1090 and 1140 cm⁻^1^ represents the contributions from overlapping vibrations of C–O stretching and asymmetric C–O–C stretching (characteristic of the PVA polymer chain), as well as the ν_3_ vibration of the phosphate group from HAp [30,48,49,50]. Moreover, the work conducted by C. Cimpeanu et al. [48] showed that in the 900–1200 cm^−1^ range, there are maxima that correspond to the typical in-plane C–H bending vibrations of aromatic compounds (such as amoxicillin).

**Figure 13:**
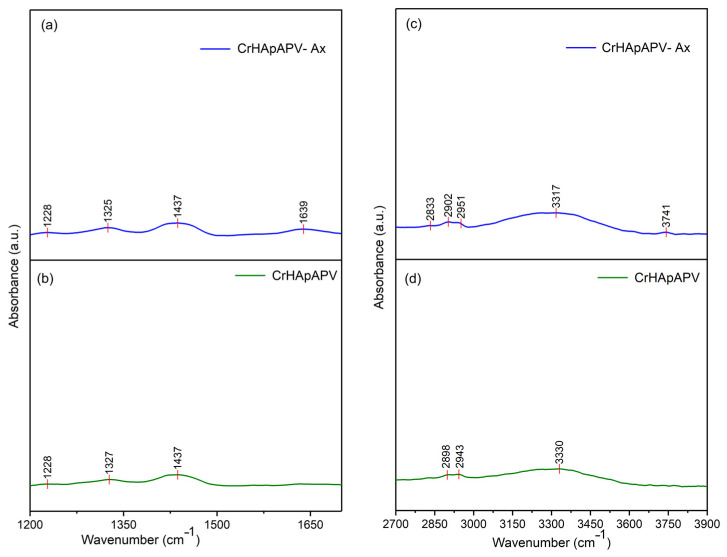
FTIR spectra of CrHApAPV-Ax in 1200–1700 cm^−1^ and 2700–3900 cm^−1^ spectral domains (**a**,**c**). FTIR spectra of CrHApAPV in 1200–1700 cm^−1^ and 2700–3900 cm^−1^ spectral domains (**b**,**d**).

In Figure 13, the FTIR spectra of CrHApAPV-Ax and CrHApAPV in the spectral regions of 1200–1700 cm⁻^1^ and 2700–3900 cm⁻^1^ are shown. Overlapping maxima appeared in these spectral domains that are characteristic of the functional groups of PVA, Hap, and amoxicillin. In the 1200–1700 cm^−1^ domain, maxima that are specific to CH_2_ group bending vibrations (from PVA) usually appear [49,50]. In the same spectral domain, there are weak maxima that could be attributed to the vibration of the β-lactam ring (at around 1772 cm^−1^), C=C stretching in the aromatic ring (at around 1616 cm^−1^), methylene (-CH_2_) groups, and amine groups (at around 1470 cm^−1^ and 1390 cm^−1^) in amoxicillin [48]. Moreover, at 2898 cm⁻^1^, 2902 cm⁻^1^, and 2943 cm⁻^1^, maxima due to the C–H stretching vibrations of aliphatic methylene (–CH_2_) groups from PVA appeared [49,50]. On the other hand, the presence of the broad maximum between 3300 and 3800 cm⁻^1^ could be attributed to O–H stretching vibrations and indicates the abundance of hydroxyl groups in the PVA backbone and HAp [49,50].

The overlap of HAp and PVA bands (in the case of CrHApAPV) and HAp, PVA, and amoxicillin bands (in the case of CrHApAPV-Ax) suggests successful composite formation, where HAp maintains its crystalline structure within the PVA matrix. Furthermore, can be observed that the addition of amoxicillin induced shifts and broadening of the maxima. Therefore, the FTIR experimental data obtained in this study are in strong agreement with previously reported results [30,48,49,50], confirming the reliability and consistency of the spectral assignments.

**Figure 14:**
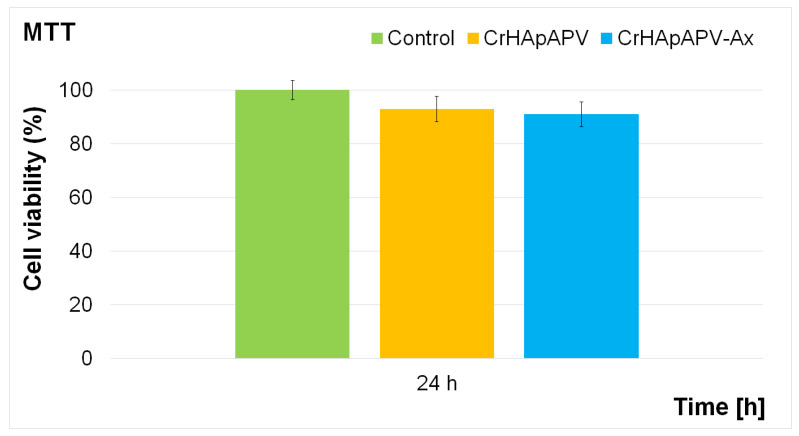
Graphical representation of the cell viability of MG63 cells exposed to the CrHApAPV and CrHApAPV-Ax coatings for 24 h. The results are depicted as the mean ± standard deviation (SD) and quantified as percentages of the control (100% viability).

The cytotoxicity of the CrHApAPV and CrHApAPV-Ax coatings was evaluated using the MTT colorimetric assay with the aid of the MG63 osteoblast-like cell line. The cell viability was measured after 24 h of incubation and is expressed graphically as the mean ± standard deviation (SD) relative to a control representing 100% cell viability. The results of the MTT assay are depicted in Figure 14. The data confirmed that both coatings exhibited good biocompatibility with MG63 cells.

The results of the cell viability assay showed that the cell viability exceeded 93% after 24 h of incubation with the CrHApAPV coating. Notably, no significant cytotoxic effects were observed for any of the tested samples. These findings indicate that the coatings provide a favorable surface for MG63 cell development by creating a good environment for cell adhesion and proliferation. The enhanced biocompatibility properties could be attributed to the presence of the PVA matrix, a known biocompatible polymer. The PVA contributed to the improved physicochemical stability and hydrophilicity of the coatings, which are essential for supporting cellular interactions and minimizing cytotoxic effects [51,52,53,54]. Similar MTT assay results were obtained for the CrHApAPV-Ax coating. The cell viability studies revealed that the presence of amoxicillin did not induce any cytotoxic effects and that the cell viability of the MG63 cells incubated for 24 h with the CrHApAPV-Ax coatings was above 91%, which is in good agreement with the ISO 10993–1:2018 standard [55] that states that materials that exhibit a cell viability rate above 88% are considered to be biocompatible, and that they will not induce significant cytotoxic responses such as apoptosis, inflammation, or immune rejection. These results suggest that the surface of the CrHApAPV-Ax coating exhibited a favorable environment for MG63 cell growth. These results are in good agreement with those of previous studies on the cytotoxicity of chromium and chromium-doped hydroxyapatite nanocomposites and coatings [30,56,57,58], highlighting the potential of these coatings in biomedical applications due to their non-toxic nature and ability to maintain high cell viability. These qualities position them as promising candidates in the future development of biomedical devices or implants. Additionally, the biocompatibility of CrHApAPV and CrHApAPV-Ax coatings, even in the early stages of MG63 cell development, emphasizes their favorable interaction with living cells, their non-cytotoxicity, and also the lack of inflammatory responses towards them. Also, our results showed that the enhanced biological properties of the CrHAp coatings could be attributed to the presence of the PVA matrix. Polyvinyl alcohol (PVA) is a synthetic polymer, which is water soluble and has been a material of interest for biomedical applications due to its outstanding biocompatibility, hydrophilicity, and chemical stability [51,52,53,54]. These characteristics confer PVA with the ability to form hydrogels that closely resemble the extracellular matrix, thus providing a favorable environment for cellular activities [51]. In addition, its ability to absorb water and swell makes it particularly effective for wound healing, drug delivery, and scaffold development in tissue engineering [52]. Moreover, although PVA has been reported to exhibit limited cell adhesion, this could be addressed through the development of composite formulations or through surface modifications. Therefore, the incorporation of calcium-reinforced hydroxyapatite (CrHAp) into PVA matrices could significantly enhance their properties such as bioactivity, osteoconductivity, and cellular interactions, particularly in bone-related applications [51,52,53,54].

**Figure 15:**
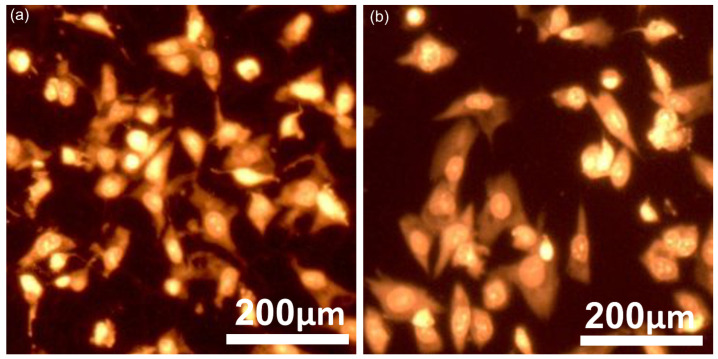
The morphology of MG63 cells grown on the CrHApAPV (**a**) and CrHApAPV-Ax coatings (**b**) visualized using fluorescence microscopy.

Information regarding the adhesion behavior and morphological characteristics of MG63 cells on CrHApAPV and CrHApAPV-Ax coated surfaces was obtained through fluorescence microscopy analysis. For these studies, the MG63 cells were cultured on both coatings, stained, and then examined under a microscope. The fluorescence imaging, depicted in Figure 15, shows that across both the CrHApAPV and CrHApAPV-Ax coatings’ surfaces, a clear and consistent pattern of cell attachment and spreading could be observed. Moreover, the images highlighted that the adhered MG63 cells exhibited well-defined, elongated morphologies with prominent actin cytoskeleton organization, thus indicating robust adhesion and presenting the signs of a healthy cellular architecture. Furthermore, the images also revealed that the adhered cells exhibited a typical fibroblastic morphology that is specific to osteosarcoma MG63 cells and they also highlight the presence of extended filopodia, which is proof of active interactions with the coating surface. Moreover, the studies emphasized that there were no signs of morphological abnormalities, like cell shrinkage, membrane damage, or nuclear fragmentation in the cells that adhered to either surface, thus demonstrating the good cytocompatibility of both coatings. These findings are in agreement with the results from the MTT assay and suggest that both CrHApAPV and CrHApAPV-Ax could provide a favorable environment that is able to promote the normal growth, adhesion, and morphological development of MG63 cells.

**Figure 16:**
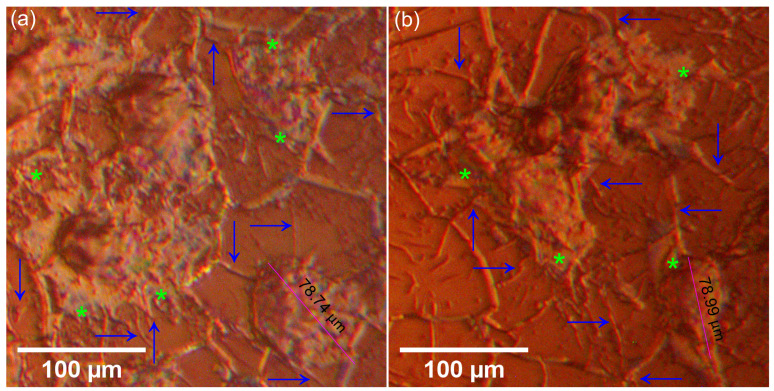
The morphology of MG63 cells grown on the surface of the CrHApAPV (**a**) and CrHApAPV-Ax coatings (**b**) visualized using metallographic microscopy after 24 h of incubation. Blue arrows mark the presence of filopodia and light green asterisks mark the presence of lamellipodia.

Additional information regarding the adherence and development of MG63 cells on the surface of the CrHApAPV and CrHApAPV-Ax coatings was gathered with the aid of metallographic microscopy. This technique was specifically used to assess the adhesion and growth of MG63 cells on the surfaces of both coatings. The metallographic images recorded under 50X magnification depicted in Figure 16 reveal that both the CrHApAPV and CrHApAPV-Ax coatings supported MG63 cell attachment and development. The images show that the adhered cells displayed a typical osteoblast-like morphology, characterized by a well-spread, elongated shape with multiple cytoplasmic extensions anchoring the cells to the coating’s surface. These results suggest that the surfaces of the coatings were a good environment for cell adherence and development. Furthermore, the images also highlighted that the cells were uniformly spread across the coating surface, and even in the early stage of development (the first 24 h), the cells had developed an interconnected network, which indicates active intercellular communication and tissue-like organization. Moreover, the metallographic microscopy images showed that there was no evidence of cellular morphological abnormalities, thus supporting the biocompatibility of both coatings.

MG63 is a human osteosarcoma cell line that is commonly used as a model for immature osteoblasts in bone biology and biomaterials research [59,60,61,62,63,64,65,66]. Morphologically, MG-63 cells are spindle-shaped and exhibit an osteoblast-like, fibroblast-like appearance, with an elongated form with various cytoplasmic extensions. In a monolayer culture, these cells adhere well to plastic surfaces and usually grow in a semi-confluent to confluent layer, showing a polygonal cell shape with intercellular contacts [59,60,61,62,63,64,65,66,67]. The MG63 cell nuclei are typically large, have an oval to round shaped, and are centrally located. Also, they exhibit prominent nucleoli, which indicates active transcription. The cytoplasm is granular and may contain vacuoles or lipid droplets, particularly during differentiation or under stress conditions. These features make MG-63 a valuable in vitro model for studying osteogenic processes and evaluating biomaterial interactions [59,60,61,62,63,64,65,66,67].

**Figure 17:**
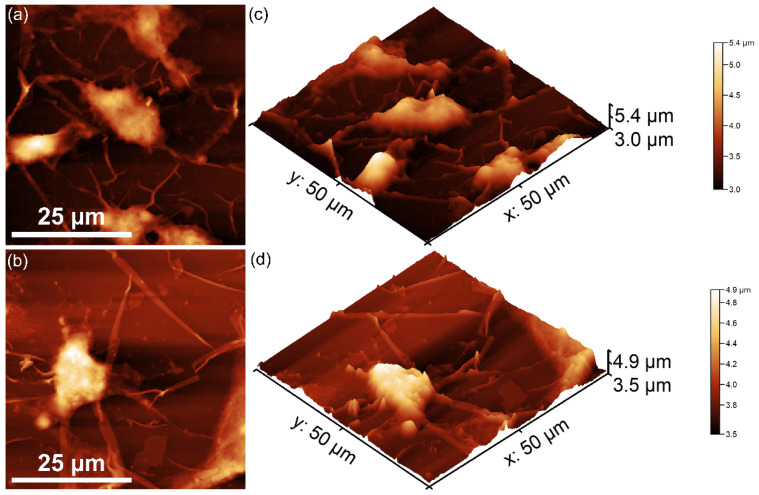
2D AFM topography of MG63 cells adhered on CrHApAPV (**a**) and CrHApAPV-Ax (**b**) coatings after 24 h of incubation and their 3D corresponding representation (**c**,**d**).

Information about the adherence and proliferation of MG63 cells on the surface of the CrHApAPV and CrHApAPV-Ax coatings was also obtained with the aid of AFM. The surface topographies of the MG63 cells adhered to the CrHApAPV and CrHApAPV-Ax coatings’ surfaces after 24 h of incubation as well as their 3D representation are presented in Figure 17a–d. The 2D AFM observations show that the cells began attaching and spreading across the surface. This could be due to the cells having an early and active interaction with the substrate. At first, the MG63 cells display a rounded morphology, but their morphology changed to a more flattened and elongated shape. These morphological transformations could be due to early cytoskeletal rearrangement and indicates that the both the CrHApAPV and CrHApAPV-Ax coatings’ surfaces promote cell adhesion. Moreover, both the 2D topographies as well as their 3D representations highlighted the presence of filopodia, which are very thin actin-rich protrusions that extend from the cell membrane and it is one of the most significant morphological indicators of cellular compatibility with a material or surface [67,68,69,70,71]. Filopodia have the ability to facilitate the initial contact between cells and a substrate, and they are also responsible for communication with surrounding cells, thereby promoting cell anchoring and signaling [67,68,69,70,71]. The observed extension of filopodia on both the CrHApAPV and CrHApAPV-Ax coatings revealed by both the MM and AFM studies strongly suggests that these materials could provide a favorable environment for cell attachment processes. In addition to the presence of filopodia, the MM and AFM results also emphasized the presence of lamellipodia, which appeared as a broad, sheet-like membrane protrusions. These results further highlight the successful adhesion and spreading of MG63 cells on the surface of the CrHApAPV and CrHApAPV-Ax coatings. The presence of lamellipodia is closely associated with cytoskeletal reorganization and active migration, and their presence typically means that the cells are not only surviving but they are also actively interacting with the material surface. The presence of both filopodia and lamellipodia on the surfaces of the CrHApAPV and CrHApAPV-Ax coatings indicates a dynamic and healthy cell morphology, which is a sign of a non-toxic environment.

The results of the AFM studies revealed that both CrHApAPV and CrHApAPV-Ax demonstrated favorable surface topography and a favorable roughness, which are known to influence cell behavior and create a favorable environment for MG63 cell adherence and development. The findings from the biological assays indicate that both CrHApAPV and CrHApAPV-Ax could provide an enhanced microenvironment for osteoblast-like cells, supporting not only initial adhesion but also sustained growth and communication.

**Figure 18:**
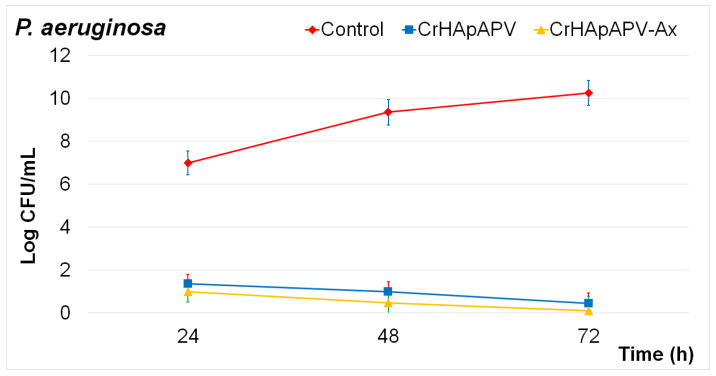
Graphical representation of the log colony-forming units (CFUs)/mL of *Pseudomonas aeruginosa* 27853 ATCC after incubation with the CrHApAPV and CrHApAPV-Ax coatings for 24, 48, and 72 h.

To further explore the biological properties of the CrHApAPV and CrHApAPV-Ax coatings, their antibacterial activity was evaluated against *Pseudomonas aeruginosa*. This bacterial strain is a Gram-negative bacterium that is responsible for infections that usually appear in the bloodstream, lungs, and other difficult-to-treat areas of the body. The antibacterial effects of the CrHApAPV and CrHApAPV-Ax coatings were evaluated in vitro over three time intervals. The results of the in vitro antibacterial assays are illustrated in Figure 18. The data are graphically represented as the mean ± SD. The findings showed a significant reduction in the number of colony-forming units (CFUs) of *P. aeruginosa* after 24, 48, and 72 h of exposure to the CrHApAPV and CrHApAPV-Ax coatings. Among the tested samples, the CrHApAPV-Ax coating demonstrated a higher antibacterial efficacy, resulting in a significantly greater reduction in CFUs compared to the control and CrHApAPV coatings. Furthermore, the data suggested that the antibacterial efficiency was influenced by the both samples as well as the incubation time.

The superior antibacterial performance of the CrHApAPV-Ax coating highlighted by the in vitro quantitative assays could be attributed to the combined effects of chromium ions, polyvinyl alcohol (PVA), and amoxicillin. The synergistic antibacterial properties of polyvinyl alcohol (PVA), amoxicillin, and chromium (Cr) ions against *Pseudomonas aeruginosa* arise from the unique combination of their individual effects, creating a complex set of antibacterial mechanisms. Amoxicillin is a broad-spectrum β-lactam antibiotic that has the ability to disrupt bacterial cell wall synthesis, which is an essential process for bacterial cell viability, proliferation, and development. By weakening the structural integrity of the cell wall, amoxicillin causes an osmotic imbalance that leads to cell lysis [72,73,74,75,76,77,78,79]. Although amoxicillin has been reported to be highly effective against numerous Gram-positive bacterial strains, its activity against *Pseudomonas aeruginosa* is limited [72,73,74,75,76,77,78,79]. This phenomenon could be attributed to the fact that this bacterial strain has a unique outer membrane that can stop antibiotic entry. Furthermore, it has intrinsic resistance mechanisms such as efflux pumps and β-lactamase enzymes that degrade the antibiotic’s β-lactam ring, rendering it ineffective. In addition, chromium, particularly in its ionic form, could induce strong oxidative stress against bacterial cells by generating reactive oxygen species (ROS) [58,80,81,82,83]. This produces damage to the bacterial cell walls and its intracellular components. Together, these components create a hostile environment for *P. aeruginosa* attachment and proliferation as they are particularly resistant due to *P. aeruginosa*’s adaptive mechanisms and robust biofilm-forming ability. While PVA itself exhibits limited or no antibacterial activity, it did show activity against *P. aeruginosa* when it was functionalized with other antimicrobial agents or nanoparticles [84,85,86]. Studies have reported that PVA composites loaded with silver nanoparticles (AgNPs), zinc oxide (ZnO), or other quaternary ammonium compounds have the ability to disrupt bacterial membranes, to generate reactive oxygen species (ROS), and impair bacterial metabolic functions, leading to cell death. The PVA matrix serves as a stable carrier that allows for controlled release of these agents, prolonging the antibacterial efficacy while reducing the cytotoxicity to surrounding tissues. In the case of the CrHApAPV and CrHApAPV-Ax coatings, the presence of PVA ensures a sustained and localized release of amoxicillin and chromium ions, enhancing their effectiveness and reducing the chances of bacterial resistance [84,85,86]. Moreover, the combination of oxidative stress from Cr, membrane disruption by Cr and amoxicillin, as well as the enzymatic interference from amoxicillin could help decrease *P. aeruginosa*’s defense systems [58,72,73,74,75,76,77,78,79,80,81,82,83,84,85,86]. This approach not only has the ability to enhance the antibacterial activity, but could also help reduce the required dosage of each individual component, potentially minimizing the side effects and cytotoxicity.

**Figure 19:**
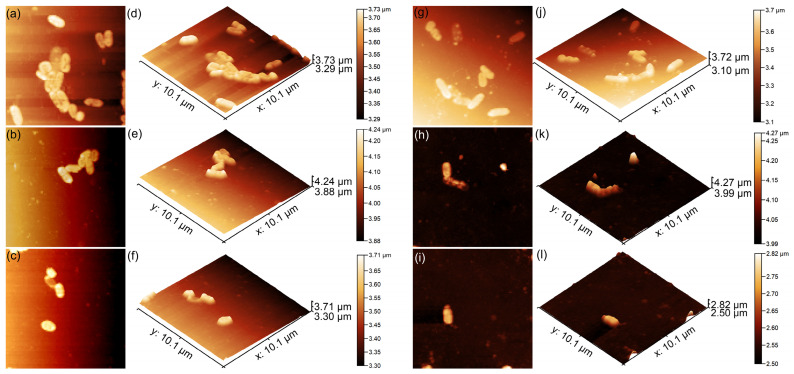
Two-dimensional AFM topography of *Pseudomonas aeruginosa* 27853 ATCC cells adhered to the surface of the CrHApAPV and CrHApAPV-Ax coatings after a 24 (**a**,**g**), 48 (**b**,**h**), and 72 h (**c**,**i**) incubation period and their 3D corresponding representations (**d**–**f**,**j**–**l**).

The attachment and proliferation of *P. aeruginosa* on the surface of the CrHApAPV and CrHApAPV-Ax coatings were also investigated using atomic force microscopy (AFM). The AFM analyses were conducted after incubating the coatings with *P. aeruginosa* suspensions for 24, 48, and 72 h under ambient conditions. The recording was performed in the non-contact mode and the scans were performed over 10.1 × 10.1 µm^2^ areas. The 2D AFM topography results as well as their 3D representations are depicted in Figure 19a–l.

The results demonstrated that both the CrHApAPV and CrHApAPV-Ax coatings effectively inhibited *P. aeruginosa* adhesion and early-stage development. Moreover, the AFM 2D images as well as their 3D representations showed that the coatings prevented biofilm formation. Furthermore, the findings showed that the CrHApAPV-Ax coating displayed enhanced antibacterial activity, suggesting a synergistic effect between chromium ions, the PVA matrix, and amoxicillin. The AFM analysis revealed a significant reduction in *P. aeruginosa* bacterial cell adherence within the first 24 h, with a significant reduction over time. After 72 h, only a few isolated cells were observed, highlighting the coatings’ long-term antibacterial efficacy. In addition, the AFM 2D surface topographies showed that even during the first 24 h of incubation, *P. aeruginosa* cells exhibited limited development on both the CrHApAPV and CrHApAPV-Ax coatings, indicating that both surfaces possess considerable antibacterial properties. The AFM analysis revealed that on the CrHApAPV coatings, the bacterial adhesion was restricted. The 2D AFM topographies highlighted that only few scattered cell microcolonies could be observed and that the bacterial cell surfaces showed early signs of stress. The presence of chromium ions contributed to this antibacterial effect by interfering with bacterial respiration and membrane integrity. Morphologically, the adhered bacterial cells appeared to be slightly deformed. Also, the surface of both coatings only appears to be moderately colonized, suggesting that the surfaces exhibited a high degree of bacterial inhibition that was sufficient to delay and inhibit biofilm initiation. On the other hand, the AFM investigations revealed that the CrHApAPV-Ax coating demonstrated higher antibacterial activity compared to CrHApAPV. The AFM imaging showed that there were very few adherent bacterial cells on the surface of the CrHApAPV-Ax coating. Also, the AFM 2D results as well as their 3D representations showed that the adhered *P. aeruginosa* cells exhibited a morphology characteristic of damaged cells, with irregular shapes and scattered debris that are indicative of cell lysis. These enhanced antibacterial properties could be attributed to the combined antibacterial action of chromium ions and the localized release of amoxicillin due to the PVA matrix, which targets cell wall synthesis.

Based on the biological studies, both the CrHApAPV and CrHApAPV-Ax coatings demonstrated good biocompatibility and a supporting and favorable environment for the adherence and development of MG63 cells. The MTT assay showed that the cells grown on both coatings exhibited a cell viability of above 91% after 24 h of incubation. Moreover, the AFM and MM studies revealed that the CrHApAPV and CrHApAPV-Ax coatings demonstrated enhanced cell attachment and proliferation properties. The MM and AFM data indicated the presence of both filopodia and lamellipodia on the surfaces of the CrHApAPV and CrHApAPV-Ax coatings, indicating that they formed a non-toxic environment and favorable environment for the adherence and proliferation of MG63 cells. These findings highlight their potential as promising candidates for use in future biomedical applications. Their in vitro antibacterial activity against Pseudomonas aeruginosa was evaluated and confirmed. The results of the antibacterial assays demonstrated that both the CrHApAPV and CrHApAPV-Ax coatings exhibited excellent inhibitory effects against P. aeruginosa bacterial cells. An AFM analysis was also used to further investigate the antibacterial activity of the coatings. The AFM findings supported the results of the quantitative assays that revealed significant antibacterial properties for the coatings. While both coatings were effective at suppressing *P. aeruginosa* colonization, the AFM results showed that the presence of amoxicillin significantly enhanced the antibacterial activity, making CrHApAPV-Ax a promising candidate for use in antibacterial devices for biomedical applications. The preliminary results on the biological properties of CrHApAPV and CrHApAPV-Ax showed that combining chromium ions, polyvinyl alcohol (PVA), and amoxicillin can offer synergistic antibacterial effects against *P. aeruginosa*, a Gram-negative pathogen known for its multidrug resistance and biofilm-forming capacity while maintaining good biocompatible properties.

## 3. Materials and Methods

### 3.1. Materials

The samples consisted of chrome-doped hydroxyapatite in a polyvinyl alcohol matrix (CrHApAPV, x_Cr_ = 0.07; [Ca + Cr]/P = 1.67) and chrome-doped hydroxyapatite in a polyvinyl alcohol matrix enriched with amoxicillin (CrHApAPV-Ax) that were produced using an adapted sol-gel method. Chromium nitrate (Cr(NO_3_)_3_·9H_2_O; Alfa Aesar, Karlsruhe, Germany; 99.99% purity), polyvinyl alcohol (PVA, [-CH_2_CHOH-]n; Sigma-Aldrich, St. Louis, MO, USA), amoxicillin (Ax, C_16_H_19_N_3_O_5_S; Sigma Aldrich, St. Louis, MO, USA), ammonium hydrogen phosphate ((NH_4_)_2_HPO_4_; Alfa Aesar, Karlsruhe, Germany), calcium nitrate (Ca(NO_3_)_2_∙4H_2_O; Sigma-Aldrich, St. Louis, MA, USA), and ethanol were used in the synthesis process without further purification. The deposition of the CrHApAPV and CrHApAPV-Ax coatings onto Si substrates was performed using the spin coating technique.

### 3.2. Development CrHApAPV and CrHApAPV-Ax Coatings

The preparation process for the CrHApAPV and CrHApAPV-Ax coatings followed the detailed methodology described in our previous study [30]. Firstly, a 0.5 mol/L solution was prepared by dissolving (NH_4_)_2_HPO_4_ in ethanol, followed by stirring at 40 °C for 2 h. In parallel, Ca(NO_3_)_2_∙4H_2_O and Cr(NO_3_)_3_·9H_2_O were dissolved in ethanol and stirred to obtain a 1.67 mol/L solution. The (NH_4_)_2_HPO_4_ solution was then gradually dropped into the Ca(NO_3_)_2_∙4H_2_O and Cr(NO_3_)_3_·9H_2_O solution under continuous stirring. The obtained mixture was then dropped into a PVA solution (10%) under vigorous stirring. The mixture was continuously stirred at 80 °C for 12 h. In the next step of the synthesis process, the mixture was washed several times with ethanol and deionized water. The same protocol was employed for the preparation of the CrHApAPV-Ax gel; the only modification was the incorporation of amoxicillin (0.2 g, 0.01 M) into the solution containing Ca(NO_3_)_2_∙4H_2_O and Cr(NO_3_)_3_·9H_2_O. Finally, the CrHApAPV and CrHApAPV-Ax gels were used to develop thin films on silicon (Si) substrates using the spin coating technique [87].

Prior to deposition, the Si substrates were pre-cleaned with acetone, followed by rinsing with double-distilled water. For each deposition cycle, 0.5 mL of the CrHApAPV and CrHApAPV-Ax gels was dispensed onto the Si substrate surface with the aid of a syringe (using a speed of 2000 rpm and a spin time of 90 s). After each deposition, the coatings were dried at 60 °C for 4 h. Finally, the CrHApAPV and CrHApAPV-Ax coatings were treated at 60 °C for 24 h.

### 3.3. Characterization Methods

This study employed non-destructive ultrasound (US) measurements to assess the stability of the CrHApAPV and CrHApAPV-Ax suspensions. Double-distilled water served as the reference medium to ensure consistent comparisons. The protocols and instruments used were the same as those described in prior studies [88], provided a robust foundation. To achieve homogeneity, each 100 mL suspension of CrHApAPV and CrHApAPV-Ax was continuously stirred at 900 rpm for 15 min at room temperature. The suspension was then transferred into a transparent cubic container outfitted with two coaxial ultrasonic transducers, spaced 16 mm apart, and positioned at the mid-height of the container box. Once stirred and prepared, the acquisition of 1000 ultrasonic signals commenced.

The structure of the CrHApAPV and CrHApAPV-Ax samples was investigated utilizing a Bruker D8 Advance diffractometer (Bruker, Karlsruhe, Germany) equipped with CuKα radiation (wavelength = 1.5418 Å) and a LynxEye™ detector; this setup ensures high efficiency and resolution. Data were collected in the angular range of 15° to 55° with 0.02° increments to provide accurate diffraction patterns. The time per step, set at 5 s, allowed for detailed peak observation.

The d-spacing of the crystal was calculated using the Bragg formula (Equation (1)).

2d_hkl_sinθ = nλ (1)

Using Equation (2), the lattice parameters were calculated:

(2) $$\frac{1}{d^{2}}=\frac{4}{3}\left[\frac{h^{2}+k^{2}+hk}{a^{2}}\right]+\frac{l^{2}}{c^{2}}$$

The unit cell volume was estimated using Equation (3):

(3) $$V=\frac{3\sqrt{3}}{2}a^{2}c$$

The average crystallite sizes were estimated utilizing Equation (4):

(4) $$D_{hkl}=\frac{K\lambda \;}{\beta_{hkl\;}cos\theta_{hkl}}$$

where *D* represents the average crystallite size, *K* is the Scherrer constant (0.94), *β* is the Full Width at Half Maximum (FWHM), (*h*, *k*, *l*) represents the Miller indices, (*a*, *c*) represents the lattice parameters, *d* is the distance between two adjacent planes (*hkl*), *θ* is the Bragg’s diffraction angle, and *λ* is the wavelength of the monochromatic X-ray beam (1.54 Å).

XPS (X-ray photoelectron spectroscopy) analysis can reveal the information about surface chemistry of samples. Using the SES 2002 instrument from Scienta Omicron (Scienta Omicron, Taunusstein, Germany) with a monochromatic Al Kα X-ray source with an energy of 1486.6 eV, precise measurements were made. The data collection adhered to earlier established protocols [89]. For processing and interpretation, CasaXPS 2.3.14 software was employed, leveraging the Shirley background subtraction method for accuracy [90]. Supplementary references from XPS tables provided additional validation [91,92]. Furthermore, charge correction was applied uniformly using the C 1s peak (at 284.8 eV) as a standard reference point for the binding energy (BE) values.

The surface morphology of the CrHApAPV and CrHApAPV-Ax coatings was investigated using scanning electron microscopy (SEM; HITACHI S4500 instrument, HITACHI, Tokyo, Japan). The elemental composition of both coatings was determined through energy-dispersive X-ray spectroscopy (EDS) analysis.

The surface topography of the CrHApAPV and CrHApAPV-Ax coatings was assessed using atomic force microscopy (AFM) (NT-MDT Spectrum Instruments, Tempe, AZ, USA). The measurements were carried out using the semi-contact mode with the help of an NT-MDT NTEGRA Probe Nano Laboratory instrument (NT-MDT Spectrum Instruments, Tempe, AZ, USA). The AFM topographies were acquired with a silicon NT-MDT NSG01 cantilever coated with a 35 nm gold layer. The surface topographies were captured over a 5 × 5 μm^2^ area. Additionally, the surface roughness (R_RMS_) values were determined for the investigated surfaces. The AFM data were processed using Gwyddion 2.59 software [93].

Furthermore, surface assessments of the CrHApAPV and CrHApAPV-Ax coatings were performed using an inverted trinocular metallographic microscope (20× magnification; MM; model OX.2153-PLM, Euromex, Arnhem, The Netherlands). The 3D MM images were obtained with the aid of ImageJ software (version 1.51j8) [94].

For the swelling test, the dried CrHApAPV and CrHApAPV-Ax coatings were first weighed (*E_d_*). Then, they were immersed in 15 mL of a PBS solution for 24 h. After 24 h, the coatings were taken, and any excess PBS solution was carefully removed using filter paper. Then, the wet specimens were weighed (*E_w_*). This process was repeated three times, and the swelling test results are presented as the mean value ± SD. The swelling percentage (%) was calculated using the following formula:

$$Swelling\;(\%)=\frac{E_{w}-E_{d}}{E_{d}}\times 100$$

Fourier-transform infrared (FTIR) spectroscopy was employed to analyze the vibrational modes of the functional groups present in the CrHApAPV and CrHApAPV-Ax coatings. Measurements were carried out using a Perkin Elmer spectrometer. For this study, the FTIR spectrometer used was equipped with a Universal Diamond/KRS-5 accessory (Perkin Elmer, Waltham, MA, USA). Then, the spectra were recorded in the 450–4000 cm⁻^1^ range at a resolution of 4 cm⁻^1^.

### 3.4. Biological Assays

#### 3.4.1. Biocompatibility Studies

The biocompatibility of the CrHApAPV and CrHApAPV-Ax coatings was evaluated by incubating them with MG63 osteoblast-like cells (ATCC CRL1427) for 24 h, according to the protocol described by Iconaru et al. [95]. The MG63 cells were cultured in DMEM supplemented with standard additives and maintained at 37 °C in a humidified atmosphere with 5% CO_2_. The coatings were placed in 24-well culture plates and seeded with 1 × 10^5^ cells per well. The cell viability was assessed after 24 h of incubation using the MTT assay. After the 24 h incubation period, the cells that had adhered to the surface were gently rinsed with phosphate-buffered saline (PBS) to remove the non-adherent cells as well as any debris. Afterwards, the cells were fixed using a 2.5% glutaraldehyde solution in PBS for 20 min at room temperature, followed by rinsing with PBS. Furthermore, a 30% ethanol solution followed by air drying were used to dehydrate the samples in order to preserve the cellular morphology and attachment on the coatings for further analysis. Fluorescence images were obtained with the aid of an Observer D1 Carl Zeiss microscope (Carl Zeiss Microscopy GmbH, Jena, Germany). Prior to examination with the fluorescence microscope, the cells were stained with propidium iodide (PI).

#### 3.4.2. Antibacterial Assay

The antibacterial properties of the CrHApAPV and CrHApAPV-Ax coatings were assessed against the *Pseudomonas aeruginosa* 27853 ATCC reference strain. The in vitro antibacterial evaluation was performed using an adaptation of the protocol of Ciobanu et al. [96] and following the standardize ISO 22196 method [97]. This method is a commonly used antibacterial assay to evaluate the activity of coatings on bacterial cells by placing a bacterial medium into direct contact with the coatings. For this purpose, a standardized bacterial suspension with an initial concentration of 5 × 10^6^ CFU/mL was prepared and brought into contact with the CrHApAPV and CrHApAPV-Ax coatings. A small volume of the prepared suspension (100 µL) was applied to each coating’s surface to ensure uniform contact. The incubation was carried out at 37 °C for periods of 24, 48, and 72 h to monitor the influence of incubation time on the antibacterial effects. The bacterial cell survival was quantified for each incubation interval and the CFU/mL values are graphically represented as log CFU/mL versus time. A positive control (C+), consisting of the bacterial suspension incubated in the absence of any coating material, was included to establish the baseline bacterial growth under the same conditions. All the experiments were conducted in triplicate to ensure reproducibility, and the results are presented as mean values and their corresponding standard deviations (mean ± SD). In addition to quantitative viability assays, atomic force microscopy (AFM) was employed to qualitatively examine bacterial adhesion and surface colonization on the surface of the CrHApAPV and CrHApAPV-Ax coatings. For this purpose, after incubation, the coatings were washed with sterile saline, fixed with cold methanol, and prepared for visualization.

#### 3.4.3. Statistical Analysis

All the data from the biological assays are presented as the mean ± standard deviation (SD). The statistical analysis was conducted using Microsoft Excel and ordinary one-way ANOVA, with the significance threshold set at *p* < 0.05.

## 4. Conclusions

The spin coating method was used to produce CrHApAPV and CrHApAPV-Ax coatings. The stability of the samples was evaluated using ultrasound measurements. This study emphasized that the stability of the suspensions is crucial for achieving successful coatings. The XRD analysis identified hydroxyapatite as the major crystalline phase in both samples, with PVA-specific peaks observed in both cases. Furthermore, the XRD analysis of the CrHApAPV-Ax sample revealed a peak specific to amoxicillin. The XPS analysis confirmed the presence of C, O, Ca, P, and Cr in both samples. Additionally, the CrHApAPV-Ax sample showed the presence of N and S, which were from the amoxicillin. The presence of the functional groups of HAp, PVA, and Ax in the analyzed samples was confirmed by the FTIR results. The SEM, MM, and AFM analyses demonstrated that the CrHApAPV coating exhibited a uniform and homogeneous surface morphology without the visible presence of cracks or fissures. Furthermore, the SEM, MM, and AFM studies also revealed that the CrHApAPV-Ax coating showed a similarly homogeneous surface but with the presence of uniformly distributed particle agglomerates. Also, the AFM data suggested that the incorporation of amoxicillin increased the surface roughness of the CrHApAPV-Ax coating compared to the CrHApAPV samples. The antibacterial studies revealed that the CrHApAPV-Ax coating was more effective in inhibiting *P. aeruginosa* cell adherence and development on its surface compared to the CrHApAPV coating. This activity is of particular importance in biomedical applications such as implantable devices, wound healing, and antibacterial and biocompatible devices. Overall, the CrHApAPV and CrHApAPV-Ax coatings could be considered promising materials for biomedical use as they can effectively promote healthy cell growth while combating bacterial pathogens.

## Figures and Tables

**Table 1:** The lattice parameters, volume of unit cell, and crystallite size.

Sample	Lattice Parameter (Å)		c/a Ratio	Volume of Unit Cell (Å)^3^	Crystallite Size (nm)
	a	c			
CrHApAPV	9.405	6.879	0.731	526.940	14.15
CrHApAPV-Ax	9.335	6.855	0.734	517.314	12.54

**Table 2:** Thickness of the CrHApAPV and CrHApAPV-Ax coatings estimated by SEM transverse cross-section studies.

Sample	Coating Thickness (nm)
CrHApAPV	175 ± 10 nm
CrHApAPV-Ax	188 ± 10 nm

## Data Availability

The original contributions presented in the study are included in the article. Further inquiries can be directed to the corresponding authors.
